# Challenges in using liquid biopsies for gene expression profiling

**DOI:** 10.18632/oncotarget.24140

**Published:** 2018-01-11

**Authors:** Tania B. Porras, Pushpinder Kaur, Alexander Ring, Naomi Schechter, Julie E. Lang

**Affiliations:** ^1^ Department of Surgery, Keck School of Medicine, University of Southern California, Los Angeles, CA, United States; ^2^ Department of Radiation Oncology, Keck Medical Center, University of Southern California, Los Angeles, CA, United States; ^3^ University of Southern California Norris Comprehensive Cancer Center, Los Angeles, CA, United States

**Keywords:** circulating tumor cells (CTCs), NanoString PAM50, gene expression, target pre-amplification, PCR

## Abstract

Circulating tumor cells (CTCs) have potential utility as a surrogate biomarker of tumor biology via a liquid biopsy. The aim of this study was to evaluate if the nCounter NanoString assay could be used for accurate gene expression profiling of CTCs using the PAM50 research-use-only CodeSet. Analysis was performed on CTCs isolated by the ANGLE Parsortix system from healthy blood spiked with the breast cancer cell lines Hs578T, SkBr3, MDA-MB-231 or MCF7. Using cell lines as gold standard positive controls and Parsortix processed blood without spiking (unspiked) as negative controls, we found an average of 12 significantly differentially expressed genes among spiked samples versus unspiked controls. We validated our findings with the NanoStringDiff differential expression statistical method. The NanoString recommended targeted pre-amplification introduced false positive results due to pre-amplification bias, and the amplification of non-cancer genes from normal leukocytes confounded gene expression profiling of CTCs. Pre-amplification bias is a concern for other similar assays that may be used as discovery tools or target validation of transcripts of interest in gene expression profiling of CTCs. We recommend the use of an unspiked negative control when evaluating CTC technologies regarding gene expression profiling. Given that the molecular profiling of CTCs as a liquid biopsy may have clinical ramifications for potential treatment selection in future clinical trials, our study emphasizes cautious consideration of pre-analytical variables such as amplification bias in the context of liquid biopsy studies.

## INTRODUCTION

Over the last years, research focused on circulating tumor cells (CTCs) has captured great attention as a potential “liquid biopsy” with recognized potential utility in cancer diagnosis, prognosis, and personalized treatments in patients with a variety of solid tumors [1]. Since CTCs are constantly released from the primary tumor and the metastatic sites into the blood circulation, their enumeration and molecular characterization provide useful “real time” information about tumor biology in the context of disease progression [2, 3]. Furthermore, different published studies have shown the scope of molecular characterization of CTCs at different levels, (DNA, RNA and protein), and functional *in vitro* and *in vivo* assays in understanding the tumor biology and the metastatic process. Similarly, CTC-based clinical studies have provided information about micrometastatic disease in early stages of cancer, patient prognosis, acquisition of resistance to therapy, risk of metastasis and disease progression. Additionally, studies also support the clinical relevance of monitoring CTC counts during treatment to evaluate treatment efficacy, and patient stratification to guide new therapy decisions and treatment management [1, 2, 4–6].

Given the low number of CTCs, cell enrichment strategies are necessary to select and capture rare CTCs from a background of millions of blood cells per milliliter. Regardless of the enrichment method, major obstacles such as CTC purity and CTC rarity pose additional challenges for CTC identification and CTC molecular characterization based on next generation sequencing, targeted multi-marker gene expression analysis by qPCR, and targeted multi-marker hybridization techniques such as the nCounter NanoString assay [7]. The CellSearch CTC assay, the only currently United States Food and Drug Administration approved system, enriches CTCs based on the detection and identification of the epithelial cell adhesion molecule (EpCAM) and pan-cytokeratins in the tumor cell membrane and cytoplasm. However, this system has limited utility enriching CTCs with low EpCAM expression, and CTC populations are recovered along with considerable amounts of contaminating leukocytes [8]. Various blood-cell-depletion techniques based on antibody selection against CD45 and cell filtration have been tested for research applications [9], but the extensive processing time and laborious sample preparations result in low CTC recovery, possible changes in gene expression due to microenvironmental conditions or assay manipulations, and reduced cell viability [10–12]. Furthermore, newly developed laboratory platforms based on cell size and cell deformability permit capturing CTCs even if cells have undergone epithelial-to-mesenchymal transition (EMT), while collecting CTCs suitable for both molecular and *in vivo* studies. A promising technology, the ANGLE Parsortix cell separation system is an epitope-independent microfluidic platform that captures and recovers viable, intact CTCs from clinical samples. The Parsortix system pumps the blood sample through a disposable cassette formed by a stepped gradient that narrows to a critical gap of 10μm. CTCs are captured at this critical gap, but smaller cells (erythrocytes and most leukocytes) pass through. Harvesting is achieved by applying a gentle reverse flow that allows for the recovery of CTCs for analysis. Various studies have shown the Parsortix system's versatility in capturing and harvesting CTCs suitable for *in vitro* culture and molecular analysis by techniques such as genomic hybridization, qPCR and RNA-seq [13–16].

The rapid decline in sequencing costs, and the advent of multiplexed gene expression assays have made it possible to perform targeted and comprehensive molecular characterization of CTCs by various molecular assays [7, 13, 17, 18]. The nCounter NanoString gene expression assay captures and counts individual mRNA transcripts using a highly sensitive multiplexed approach [19]. The Prosigna assay (NanoString Technologies, Seattle, WA) is an *in vitro* diagnostic platform utilizing the NanoString technology which has been accepted into certain international treatment guidelines based on clinical validation of the prognostic significance of this gene expression based assay [20]. The NanoString PAM50 assay has been optimized to classify tumor material from formalin-fixed, paraffin embedded (FFPE) breast cancer as intrinsic subtypes that can serve as a prognostic indicator for late distant recurrence-free events (>5 years after treatment) in early stage hormone positive breast cancer patients [21, 22]. The nCounter NanoString PAM50 analysis system additionally allows the direct detection, in a single reaction, of the quantity of transcripts from 50 specific endogenous genes and from 8 different reference genes using a pair of mRNA target-specific multiplexed CodeSet probes (capture and reporter probes). After hybridizing the target sample with the probe pairs, the abundance of mRNA transcripts is digitally counted by the number of times a color-coded probe is detected. Counts are subsequently used to measure gene expression levels [19, 23].

Although this multiplexed 50-gene test has been validated on FFPE breast tumor tissue, in the present study we aimed to evaluate the feasibility and accuracy of the NanoString nCounter platform for CTC-gene-expression analysis using the PAM50 Research-Use-Only CodeSet following the standard nCounter single cell gene expression protocol.

## RESULTS

### ANGLE Parsortix system allows CTC recovery

**Figure 1:**
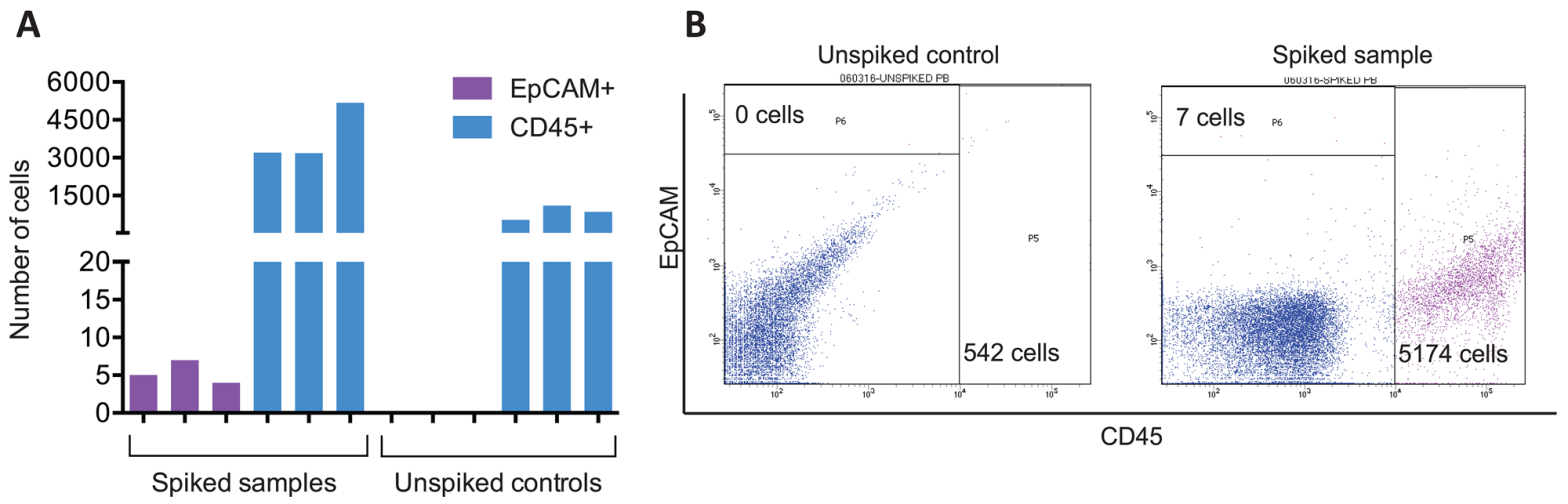
Parsortix system recovers CTCs from spiked samples **(A)** Number of EpCAM+ and CD45+ cells recovered using Parsortix system quantitated by FACS. Results from three independent SkBr3 spiked blood samples and unspiked controls. EpCAM+ cells correspond to recovered SkBr3 cells, and CD45+ cells account for white blood cell carryover. **(B)** Representative FACS results from EpCAM and CD45 staining on Parsortix harvested material.

As a proof-of-principle experiment, we determined the CTC enrichment capability of the ANGLE Parsortix system by measuring the CTC recovery rate or the number of harvested CTCs after processing SkBr3 spiked blood samples (these samples were not subjected to NanoString PAM50 analysis). CTC recovery rate was calculated by SkBr3-EpCAM+ cell enumeration by flow cytometry. By quantitating the number of endogenous WBCs CD45+ carryover cells, potential leukocyte contamination was also assessed. FACS results indicated that the Parsortix system recovered an average of 5 EpCAM+ cancer cells (20-35%) from blood samples initially spiked with 20 SkBr3 cells (Figure 1). Although it has been previously shown that the Parsortix system recovers about 40-80% of CTCs [8, 14], the relatively low number of the identified EpCAM+ captured cells may be explained by heterogeneity in EpCAM expression among SkBr3 cells, as well as cell loss during the antibody staining, washing and FACS process. Average leukocyte carryover was 2342 CD45+ cells (range 542-5174 cells) among all spiked samples and unspiked controls. Furthermore, we also spiked MDA-MB-231 GFP expressing cells in healthy donor blood, and found that the CTC capture efficiency of the Parsortix system (measured as the number of GFP positive cells trapped in the critical gap of the Parsortix cell separation cassette) ranged between 61-75% (data not shown).

### Targeted gene pre-amplification is required for NanoString PAM50 assay

**Figure 2:**
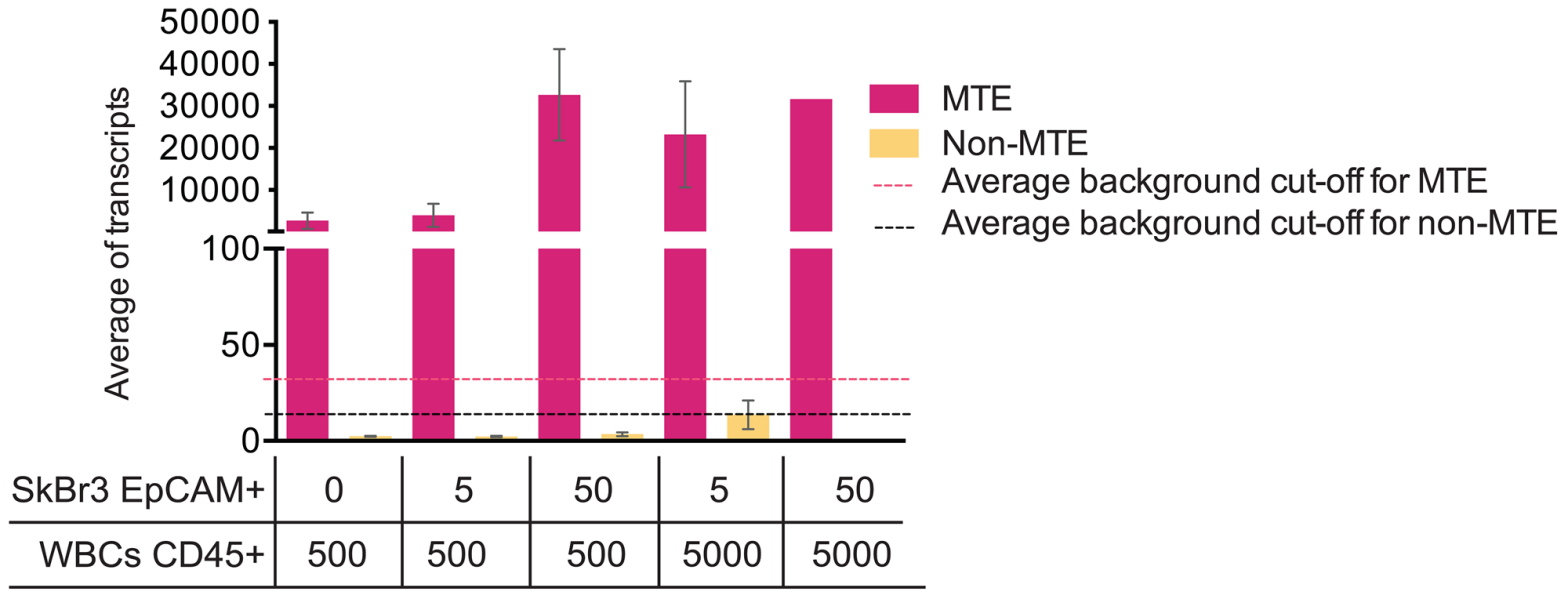
MTE influences NanoString PAM50 transcript counts Average of PAM50 transcript from MTE and non-MTE cell lysates obtained from EpCAM+ and CD45+ FACS sorted cells. SEM (Standard Error of the Mean) is plotted along with the average of background cut-off (dotted lines) for both MTE and non-MTE samples calculated using internal negative controls following the NanoString normalization protocol.

Next, we sought to test if multiple target enrichment (MTE) is a necessary step when performing NanoString analysis on Parsortix harvested material. Therefore, we created five scenarios in which different ratios of FACS sorted SkBr3 EpCAM+ cells and CD45+ cells were mixed and lysed. Gene expression analysis by NanoString PAM50 was performed on both pre-amplified (MTE) and non-pre-amplified (non-MTE) products from the same cell lysate. Results in Figure 2 show very low numbers of transcript copies, below average background cut-off level (14 counts, range between 10-22 counts) on non-MTE samples compared to MTE samples (average background cut-off of 32 counts, range between 18-54 counts). These results indicate that in order to obtain robust PAM50 gene signal on Parsortix CTC harvests, the recommended target pre-amplification is required. Therefore, we performed MTE amplification on the Parsortix harvests from all unspiked controls and from all spiked samples included in this study.

### nSolver gene expression analysis of CTC mimic samples using NanoString PAM50 assays

To identify a specific CTC PAM50 gene signature among blood samples spiked with breast cancer cell lines, we performed a differential NanoString PAM50 gene expression analysis to discriminate gene expression profile between paired blood samples: spiked and unspiked.

**Figure 3:**
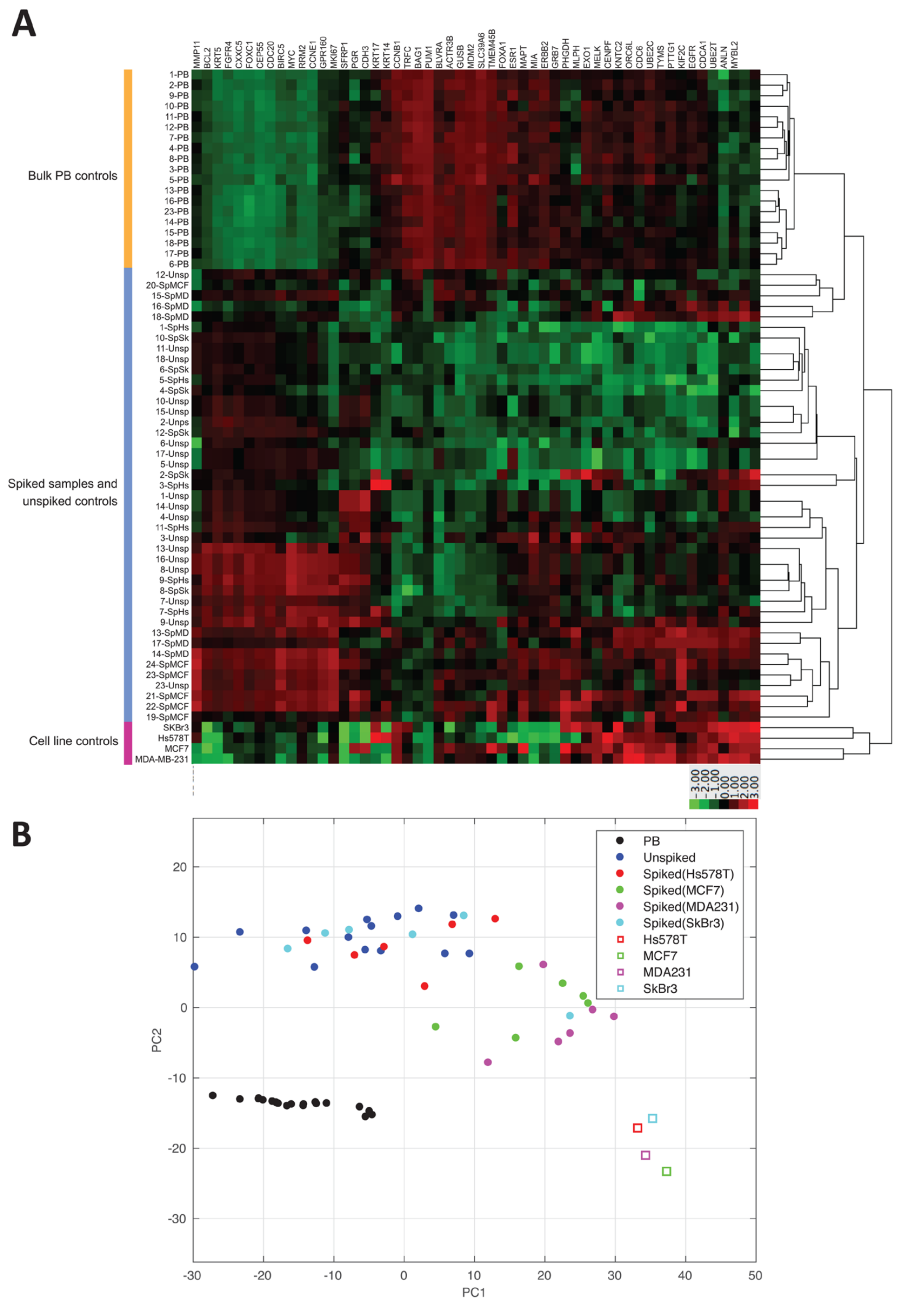
NanoString PAM50 poorly discriminates spiked samples from unspiked samples **(A)** Hierarchical clustering of NanoString PAM50 counts among all samples shows gene expression from 50 endogenous genes and 4 reference genes normalized against PSMC4, RPLP0, SF3A1 and MRPL19. Clustering was performed by average linkage method using the Euclidean distance measure considering z-score of 3 and -3 based on reference gene expression. PB: bulk peripheral blood, SpHs: blood spiked with Hs578T, SpSk: blood spiked with SkBr3, SpMD: blood spiked with MDA-MB-231, and SpMCF: blood spiked with MCF-7. **(B)** Principal component analysis of normalized gene expression data of all spiked samples, unspiked controls, bulk RNA, and cell line controls.

nCounter NanoString PAM50 data was normalized using nSolver Data Analysis Software. Intriguingly, we observed differences in gene expression of reference genes among spiked samples and unspiked controls. Among the 7 NanoString PAM50 reference genes included in the MTE primer pool (Table 1), only PSMC4, RPLP0, SF3A1 and MRPL19 were constitutively expressed among all spiked samples, unspiked controls and cell line controls. Therefore, for further differential gene expression analysis we re-normalized NanoString PAM50 counts against those 4 reference genes. By performing a hierarchical clustering of the re-normalized transcript counts, we found poor distinction between spiked samples and unspiked controls, but clear distinction between unspiked samples and bulk PB control samples (Figure 3A). Similarly, results showed a wide distribution among spiked samples and unspiked controls, but clear correlation among PB controls by the principal component analysis as shown in Figure 3B. These results suggest that NanoString PAM50 CodeSet panel not only targets cancer specific genes, but also gene transcripts expressed by normal blood cells confounding gene expression of enriched CTCs when compared to control samples.

**Table 1:** List of NanoString PAM50 MTE primers

Gene name	Forward sequence	Reverse sequence
ACTB^*^	Unable to design primers around probe region	
**ACTR3B**	ATGTGGATCCCCGGAAGT	TCGATGGGGCAGTTCTGT
**ANLN**	GAACCACCGTTTCCATCG	AGAGAGGGGCTGCTGGTT
**BAG1**	AGGAGGTGACCAGGGAGG	TCCAAGTGCTGACAACGG
**BCL2**	CTCCTGTCTTTGGAAATCCG	AACACCTTCTCCCCAGCC
**BIRC5**	CAGAGTCCCTGGCTCCTCT	GGCTCACTGGGCCTGTC
**BLVRA**	AACTGTGGGAGCTGGCTG	TCCCCAAAGAGGGAGACC
**CCNB1**	TGGATGCAGAAGATGGAGC	GGTCTCCTGCAACAACCTG
**CCNE1**	TACTTGCTGCTTCGGCCT	TCCAAGCTGTCTCTGTGGG
**CDC20**	CAGCAGCAGAAACGGCTT	GGCCAAATGTCGTCCATC
**CDC6**	TGCTCTTGATCAGGCAGTTG	TGTCAAACGGGTTTCCTTG
**CDH3**	CCAGACAGTGGGCAGGTC	GATGGTGATCTGACGGGG
**CENPF**	GCCTGGCTGCACAGAAGT	ACCCTCAGGCCCTCTCTG
**CEP55**	CGAGTCCTTGAGGCTGAGA	TCGTGAGGTTGCAGCAGA
**CXXC5**	GGTGGTTTCAGTGACGGC	TGCTAGGGACGTGGAGATG
**EGFR**	TACTTGGAGGACCGTCGC	TCACCCCGTAGCTCCAGA
**ERBB2**	ACACCTAGCGGAGCGATG	TCTTTGTTGGCTTTGGGG
**ESR1**	ATGGAGCACCCAGGGAA	CTTCAGGGTGCTGGACAGA
**EXO1**	GGGGAAAGTCTCGGAAGC	GCCAAAAGCTAGGAGATCCG
**FGFR4**	GGAGCTGCTGTGCAAGGT	GTCCTCGGCTGACACGTT
**FOXA1**	AGCTGCTCAGCTCCCCTC	GGAGTTCATGGAGCCCAG
**FOXC1**	GCCAGCAGCAGAACTTCC	AACTTGCTACAGTCGTAGACGAAA
**GPR160**	CCAAGCTTTCATTTAAGTGTCAAA	CCATGAAAAATGACAGCCAG
**GRB7**	CAGGACGGAAGCTTTGGA	TTTCGAAGCTTGTTGGGC
GUSB^*^	CCACATGCAGGTGATGGA	ATACGGAGCCCCCTTGTC
**KIF2C**	GGAGAACCAAGCATTCTGC	TGGATGCATTCTGGGCTT
**KRT14**	GCAGGAGATCGCCACCTA	GGCAGCCTCAGTTCTTGG
**KRT17**	TGCTGGAGGGAGAGGATG	CCGGGGTAGCTGAGTCCT
**KRT5**	CTCTCCAGCACCTCCCAA	ACGGAGGTGAAGCTGGTG
**MAPT**	CCCCTCTTTGGGAGAGGA	TGTTTGGGGCTAAGGCAA
**MDM2**	CGTGAAGGAAACTGGGGA	TTTTTGTGCACCAACAGACTTT
**MELK**	ACGGAGATTGAGGCCTTG	TCCCTGTGAGCATAGCCC
**MIA**	TGCCGATTCCTGACCATT	CGGACAATGCTACTGGGG
**MKI67**	CCAGTGATCAACGCCGTA	CATCTGCCCATGATTTTGC
**MLPH**	GTGAAGCCCTCGGGAAAG	GCGAGCCTCGGTACACTG
**MMP11**	TGACCAGGGCACAGACCT	CCTGGAGGTGACAGTGGG
MRPL19^*^	GGAGAAAAGTACTCCACATTCCA	CTCCTGGACCCGAGGATT
**MYBL2**	TCTTGCTGGACCGAGGAG	GGGCTTGCAGTCTTTGGA
**MYC**	AACCGAAAATGCACCAGC	CTCCTCTGCTTGGACGGA
**NAT**	Unable to design primers around probe region	
**NDC80 (KNTC2)**	GTCCCTGGGTCGTGTCAG	TTTCTCTTTGGTTTGAGGGG
**NUF2 (CDCA1)**	CCTGCAGACAGACGCCTT	GGTTTTTACCATCAGCTCCTGT
**ORC6**	AATGGTAGCCACATCCGGT	TTCTCCATTTCCTTTGCTGG
**PGR**	TCAGAAGCCAGCCAGAGC	CTAAGGCGACATGCTGGG
**PHGDH**	TGCCGCAGAACTCACTTG	CTGGGGAAATGATGGGGT
PSMC4^*^	CAGGAGGAGGTGAAGCGA	GCATTGCTGTGCTTGTGG
**PTTG1**	CAACACCACGTTTTGGCA	GGCATCATCTGAGGCAGG
PUM1^*^	TCCTGGGTGATCAATGGC	CTTGGGCCAAATCCTCCT
RPLP0^*^	GCGACCTGGAAGTCCAAC	TGTTTTCCAGGTGCCCTC
**RRM2**	CGCCGCTTTGTCATCTTC	GGCTAAATCGCTCCACCA
SF3A1^*^	CTTCTTGACCCTCGCTGG	TGGATCTCCTCCTCACCG
**SFRP1**	CCCACCTTTCAGTCCGTG	ATGGCTACCCTGGGGTTT
**SLC39A6**	GGAGATTAAGAAGCAGTTGTCCA	ATTCTTGCACCCTCTGGG
TFRC^*^	GGACATGCTCATCTGGGG	TCTGTTTTCCAGTCAGAGGGA
**TMEM45B**	CACCTTTTGGAACACCCG	CAAGAGGGCGGTCTGGTA
**TYMS**	GGAAGGGTGTTTTGGAGGA	CCCAAAATGCCTCCACTG
**UBE2C**	CCTGCTATCACCCCAACG	CTGGCTGGTGACCTGCTT
**UBE2T**	TGGTACCCCGTTGGTCC	GGGTGGCTCTGTGGCTAA

^*^ Reference genes.

**Figure 4:**
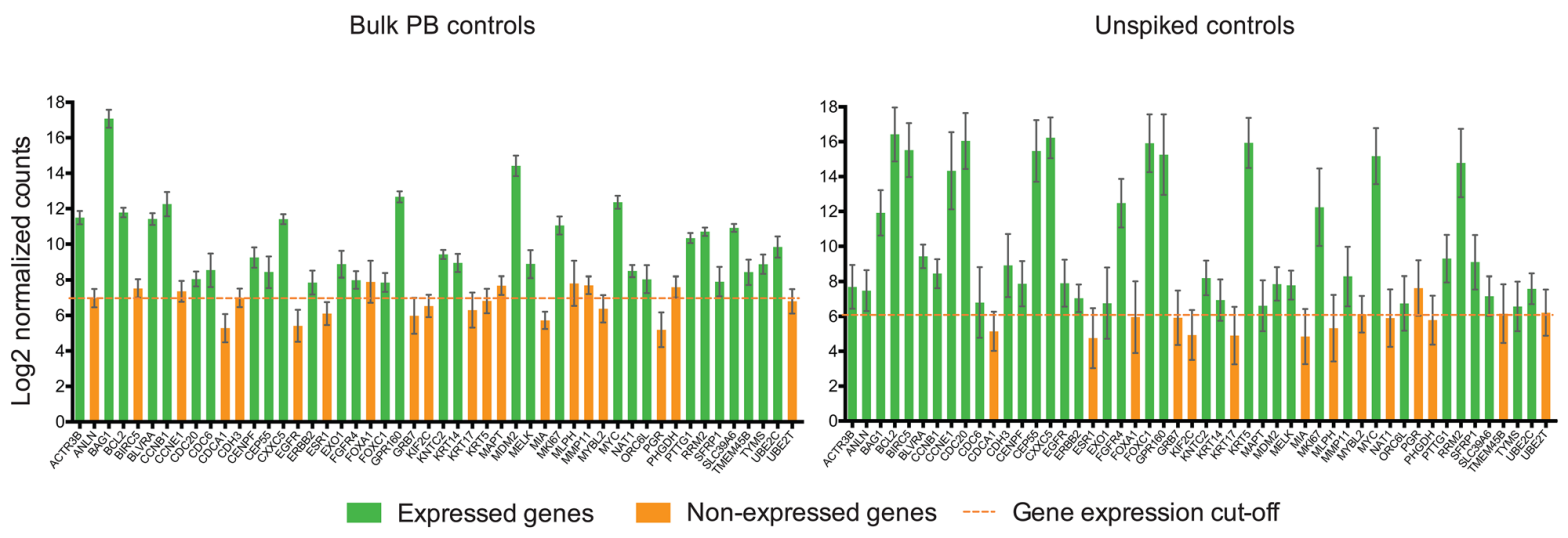
MTE introduces amplification bias to NanoString PAM50 counts Bar plots show the average of the Log2 gene expression of PAM50 endogenous genes for all bulk PB controls (n=19) and for all unspiked samples (n=24). Error bars represent the SEM, and horizontal dotted line corresponds to the gene expression threshold or cut-off background level. NAT gene is not plotted in unspiked controls due to absence of MTE primers.

Additionally, we compared the gene expression among bulk PB controls (RNA extracted from blood, but not subjected to MTE) versus unspiked blood controls (subjected to MTE). We not only observed relatively high gene expression of some genes by unspiked controls compared to bulk PB control (Figure 4), but genes such as ANLN, BIRC5, CCNE1, CDH3, EGFR, KRT5, MAPT, and MMP11 were found to be expressed in unspiked samples, while not expressed by bulk PB controls, suggesting that pre-amplification during MTE cycles may produce false positive gene expression results. Since the MTE step was performed only on the NanoString input material, the performance of the internal negative controls used to establish that the background expression threshold was not altered. In fact, similar cut-off values were obtained as shown in Figure 4. On ANOVA testing, by comparing the mean log of normalized counts among all bulk PB controls versus all unspiked samples, we found that 41/49 (83.7%) of the PAM50 genes were significantly different (*p*<0.001).

Furthermore, transcript counts for individual genes between bulk PB controls and unspiked samples among different donors did not reflect the linear unbiased target pre-amplification expected after the MTE process. We observed inconsistent transcript-count-fold changes between unspiked controls and bulk PB controls among genes and among healthy donors (Table 2). For example, by comparing fold change values for reference genes among all donors, we observed that some of them, such as PSMC4, PUM1, RPLP0 and SF3A1 were over-amplified in donor 6 compared to those in other donor samples (fold changes ranging from 0 to 102). We noticed similar discordant fold-change results among those 8 genes with opposite expression between unspiked samples and bulk PB controls (Table 2). These discrepancies not only support our finding of the inconsistent biased amplification during the MTE step, but also reflect normal patient-to-patient heterogeneity. Furthermore, a non-template control (PBS) subjected to MTE in three technical replicates showed expression of two genes: KRT17 and MYC.

**Table 2:** Transcript count-fold change of unspiked controls versus bulk PB controls among all 19 different healthy donors

		Donor 1	Donor 2	Donor 3	Donor 4	Donor 5	Donor 6	Donor 7	Donor 8	Donor 9	Donor 10	Donor 11	Donor 12	Donor 13	Donor 14	Donor 15	Donor 16	Donor 17	Donor 18	Donor 19
Reference genes	ACTB	−1	−1	−1	−1	−1	−1	−1	−1	−1	−1	−1	−1	−1	−1	−1	−1	−1	−1	−1
	GUSB	−1	−1	−1	−1	2	−1	−1	−1	−1	−1	−1	−1	−1	−1	−1	−1	0	−1	−1
	MRPL19	0	0	−1	−1	21	0	0	0	0	0	0	−1	−1	0	−1	−1	3	−1	0
	PSMC4	2	12	−1	0	102	25	4	0	−1	6	7	0	0	−1	1	−1	11	2	0
	PUM1	−1	0	−1	−1	35	0	0	−1	−1	−1	0	−1	−1	−1	−1	−1	5	−1	0
	RPLP0	1	4	−1	0	55	8	3	3	1	4	6	0	3	0	0	0	14	3	0
	SF3A1	−1	0	−1	−1	24	2	1	0	−1	0	0	0	−1	−1	−1	−1	7	0	−1
	TFRC	−1	−1	−1	−1	1	−1	−1	−1	−1	−1	−1	−1	−1	−1	−1	−1	0	−1	−1
Amplified genes	ANLN	0	2	0	−1	10	15	3	2	3	1	2	−1	1	−1	0	0	2	1	0
	BIRC5	114	448	5	23	7165	500	1843	1304	456	350	273	76	1088	61	74	638	1432	140	396
	CCNE1	41	269	2	13	17722	262	1822	1919	296	55	100	60	169	8	12	339	1181	72	203
	CDH3	43	7	2	2	16	10	6	2	3	6	1	1	3	10	3	1	3	1	1
	EGFR	126	15	0	3	39	5	5	8	14	14	1	0	8	6	1	2	8	1	1
	KRT5	2086	6892	68	169	4460	1240	1108	715	1112	1225	379	35	1797	473	524	402	1114	197	375
	MAPT	−1	0	−1	0	1	0	0	1	−1	0	−1	−1	1	−1	−1	0	0	−1	−1
	MMP11	1	1	−1	−1	21	0	7	6	1	4	4	−1	0	−1	0	0	1	3	6

**Figure 5:**
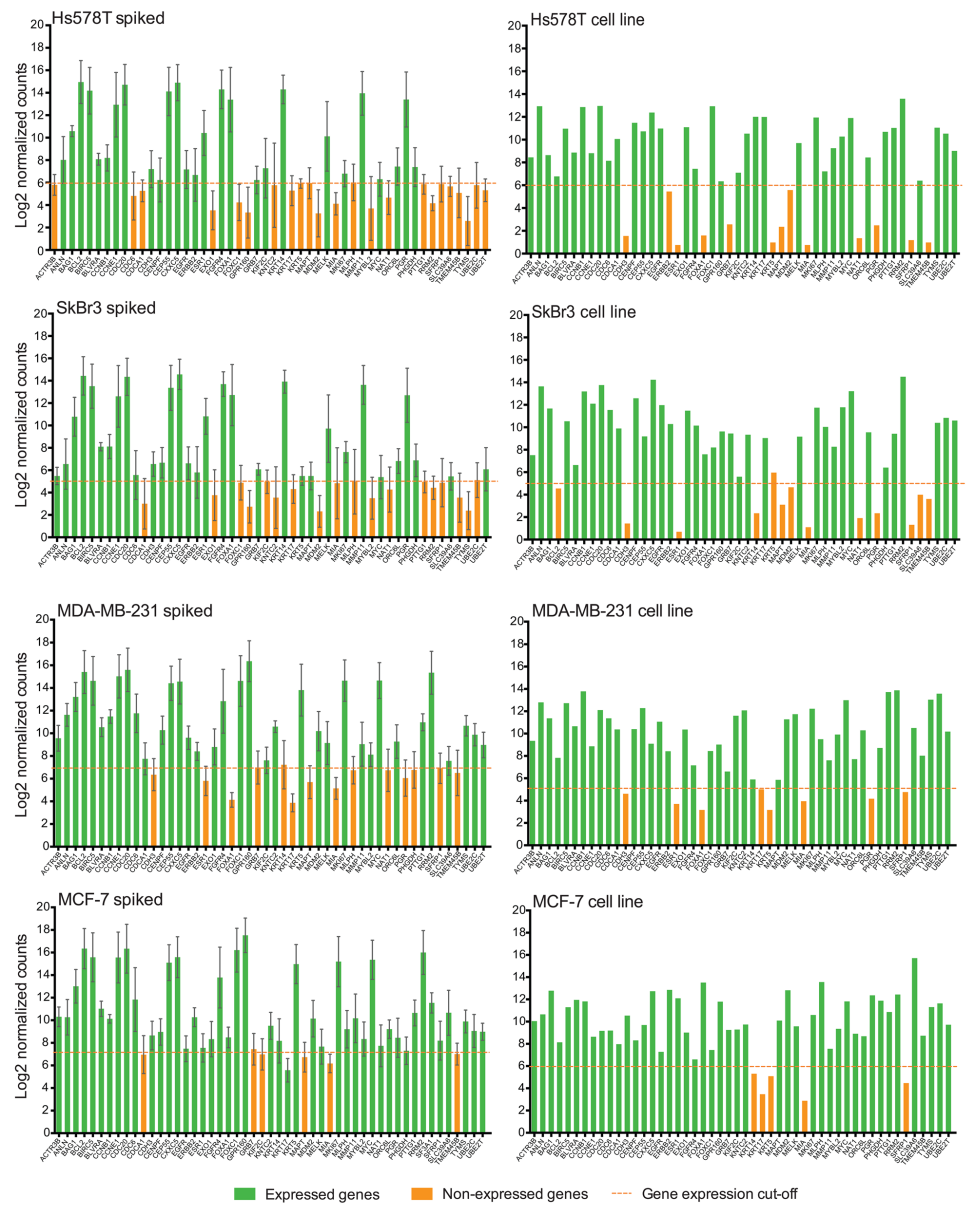
NanoString PAM50 may not distinguish CTC gene signature Bar plots show the average of the Log2 gene expression of 50 endogenous genes for all spiked samples (n=6 for each cell line), and for cell line controls. Error bars represent the SEM, and horizontal dotted line corresponds to the gene expression threshold or cut-off background level. NAT gene is not plotted in spiked samples due to absence of MTE primers.

Conversely, when performing a global comparison of NanoString PAM50 gene expression among spiked samples with the corresponding cell line control (Figure 5), we found a few downregulated genes (1-7 genes per cell line) which expression correlated between samples and cell line controls. In Hs578T spiked samples, KRT5, MAPT, MDM2, MIA, SFRP1 and TMEM45B; in SkBr3 spiked samples, MDM2, MIA, SFRP1 and TMEM45B; in MDA-MB-231 spiked samples, CDH3, ESR1, FOXA1, KRT17, MIA, PGR and SFRP1; and in MCF-7 spiked samples only MIA were found to be similarly under-expressed among spiked samples and cell line controls. In contrast, we found differences in gene expression in almost half of the PAM50 genes between spiked samples and controls (Figure 5), particularly genes such as CDCA1, CDH3, ESR1, EXO1, FOXA1, GPR160, KNTC2, KRT17, MLPH, MYBL2, PGR, PTTG1, RRM2, SLC39A6, TYMS, and UBE2C had opposite findings on spiked samples with each cell line versus the corresponding bulk cell line regarding the assay calling expression versus no gene expression signal. These findings in conjunction with the difficulty in obtaining ultra-pure CTC enriched populations raise the concern that amplified expression of background leukocytes may affect nCounter transcript counts as well as the resulting importance of selecting genes that are not expressed in normal peripheral blood cells.

### Differential gene expression in breast cancer-cell-spiked samples

**Figure 6:**
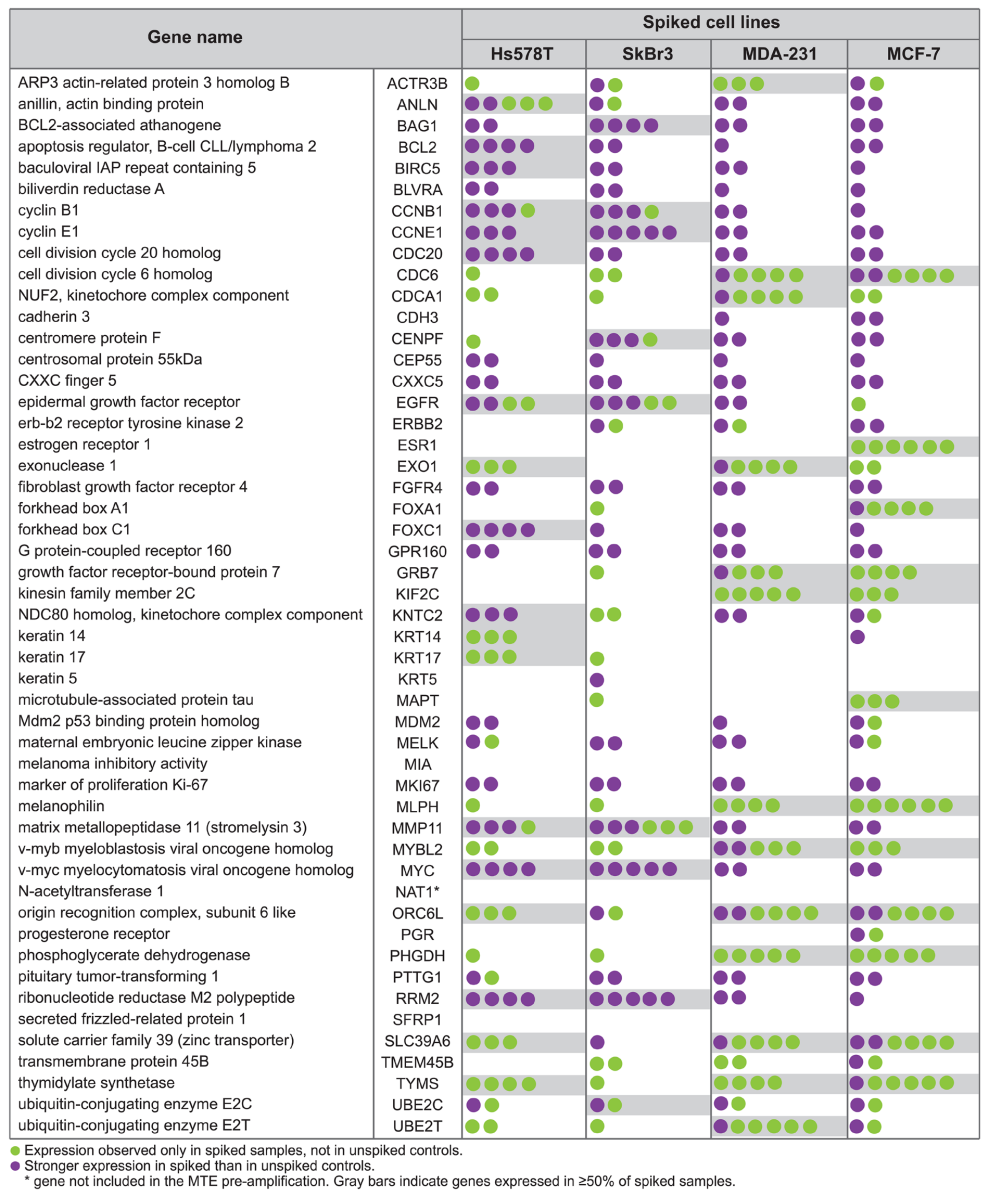
Differentially expressed genes between spiked samples and unspiked controls Each colored dot represents a comparison between the spiked sample and the unspiked control for each individual healthy donor.

To determine a unique set of genes differentially expressed among spiked samples that could be pinpointed as a tumor specific gene signature, we performed a gene expression selection based on the background cut-off value, and using corresponding unspiked samples and cell lines as negative and positive controls, respectively. As shown in Figure 6, differentially expressed genes were classified in three main groups: (i) genes only expressed in spiked samples and in cell line controls but not expressed in unspiked controls (green dots), (ii) genes expressed by all spiked, unspiked and cell lines, but with stronger expression in spiked samples compared to unspiked control (purple dots). For this group, we defined stronger expression as 20 or more transcript counts over the unspiked control, and (iii) not expressed genes in neither spiked samples nor in unspiked and cell line controls (no dots). Based on this classification, we found an average of 12 genes either uniquely expressed or highly expressed in ≥50% of the 6 samples spiked with the different cell lines. Specifically, Figure 6 shows that 18, 9, 13, and 12 genes (gray bars) for Hs578T, SkBr3, MDA-MB-231 and MCF-7, respectively, were considered as potential cell line specific genes. This demonstrated that there is high variability in NanoString gene expression of both Parsortix CTC mimic samples (spiked samples), and Parsortix processed negative expression controls (unspiked) among donors.

Since those differentially expressed genes correspond to a very small fraction of the genes included in the NanoString PAM50 panel, our results indicated that the data normalization and the background correction performed by the nSolver Analysis software during gene expression analysis of nCounter data may have limited utility pinpointing differentially expressed genes that correspond to unique tumor cell gene signature in CTC mimic samples versus leukocyte background.

Furthermore, we evaluated the potential diagnostic accuracy of the NanoString PAM50 on our breast cancer CTC-mimic-model for future use as validation technique for other assays such as RNA-seq and microarrays. By comparing PAM50 gene expression results from spiked samples to those from the corresponding cell line gold standard control we were able to test the ability of the NanoString PAM50 to predict and to classify Parsortix spiked samples as truly CTC specimens. The low sensitivity (<50%) and specificity (<75%) detecting cell line gene signatures in spiked samples, in conjunction with the relatively high false positive rate (60-80%) shown in Table 3, indicate that the leukocyte gene amplification, and the over-amplification of some genes after MTE confound CTC gene expression posing major obstacles for using NanoString PAM50 in Parsortix CTC enriched populations to monitor tumor biology in breast cancer.

**Table 3:** Diagnostic performance for CTC mimics (n=6) versus cell line control

			Spiked cell lines	
	Hs578T	SkBr3	MDA-231	MCF-7
Sensitivity	0.50	0.47	0.43	0.44
Specificity	0.34	0.17	0.74	0.40
False negative rate	0.50	0.53	0.57	0.56
False positive rate	0.66	0.83	0.26	0.60

**Figure 7:**
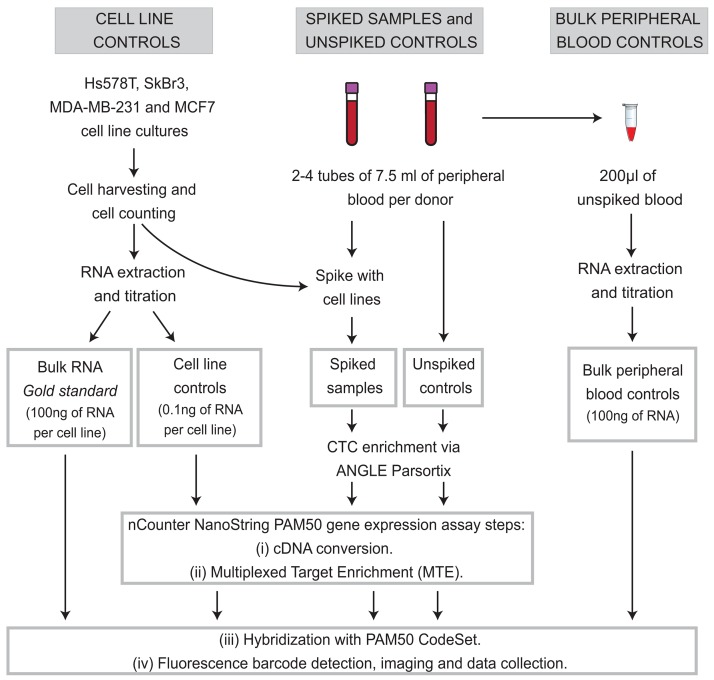
Experimental workflow Two peripheral blood specimens from 19 different healthy donors were collected, one spiked with tumor cells served as a CTC mimics (spiked samples, n=24. Blood from donors who donated more than 2 tubes of blood were spiked with more than one cells line), and other samples without adding tumor cells (unspiked control, n=24) were used as CTC negative control. RNA from cultured cells lines (0.1ng of extracted RNA) was used as positive control for gene expression for spiked MTE samples, and bulk RNA from the same cell lines (100ng) was used as ‘gold standard’ for NanoString PAM50 gene expression analysis. Bulk RNA from peripheral blood controls (no Parsortix processing) was also used as a negative control for gene expression.

**Figure 8:**
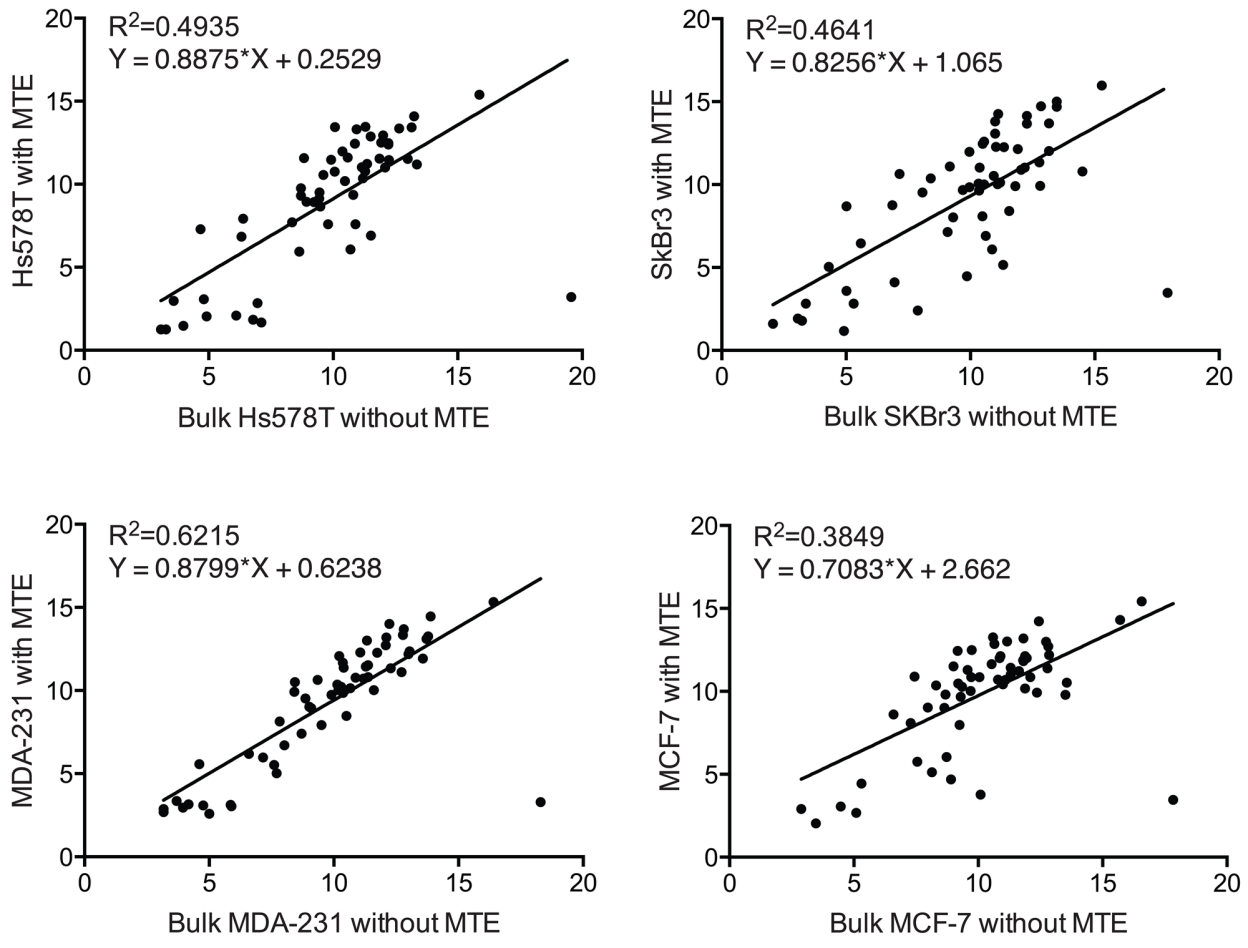
Pairwise correlation between MTE and non-MTE cell line controls Scattered plot and Pearson's correlation analysis between Log2 of normalized NanoString PAM50 transcript counts of bulk cell line RNA (without MTE) and cell line RNA (with MTE) for all genes included in the PAM50 panel.

Additionally, we performed a pairwise correlation and linear regression analyses on the PAM50 gene expression between cell line controls and bulk cell line RNA. Since bulk cell line RNA was not subjected to MTE (100ng of extracted RNA as NanoString input material) as shown in Figure 7, we used its NanoString PAM50 gene signature as a gene expression ‘gold standard’. As indicated by the scatter plots and the coefficients of determination in Figure 8, less than 62% of gene expression correlated (R^2^=0.49, R^2^=0.46, R^2^=0.62, and R^2^=0.38 for Hs578T, SkBr3, MDA-231, and MCF-7 controls respectively) between MTE cell line control versus non-MTE ‘gold standard’ cell line RNA.

### Differential gene expression validation using NanoStringDiff statistical method

To validate our differential gene expression analysis, we used the open source statistical platform NanoStringDiff [24]. Following the described protocol, we used the NanoStringDiff script to identify and/or validate differentially expressed genes among spiked samples. Briefly, this method assumes a negative binomial-based model to fit the discrete nature of the nCounter data and corrects for platform source of variation, sample content variation and background noise. Additionally, *q*-values are determined as a refined level of significance for every comparison per gene. While *p*-values measure significance in terms of false positive rate, the *q*-values measure it in terms of false discovery rate (FDR). *p* and *q*-values <0.05 were considered significant.

In Table 4, we show all *p* and *q*-values for the NanoStringDiff comparison analysis between each pair of spiked sample and unspiked control per donor. We found that only 8, 2, 21, and 17 genes (bold font) were statistically significantly expressed in Hs578T, SkBr3, MDA-231, and MCF-7 spiked samples, respectively. By performing the NanoStringDiff statistical method, we could validate some of the differentially expressed genes previously found after nCounter normalization and gene expression analysis. From the differentially expressed genes shown in Figure 6, NanoStringDiff validated 5 out of 18 genes among Hs578T spiked samples; 11 out of 13 genes in MDA-231 spiked samples; and 7 out of 12 of the genes among MCF-7 samples. Differentially expressed genes in spiked samples confirmed by both nCounter and NanoStringDiff analyses are highlighted by the gray boxes in Table 4. Although no standard method has been proposed for differential gene expression analysis on nCounter data from liquid biopsies, NanoStringDiff may be considered as an alternative statistical method allowing for multivariate analysis to select independent genes that define the outcome of sample type. However, we emphasize the need for a standardized methodology for gene expression analysis of nCounter NanoString PAM50 data when used for targeted transcriptome profiling of CTC or as validation technique of more comprehensive analysis such as whole transcriptome sequencing RNA-seq.

**Table 4:** Differentially expressed genes between spiked and unspiked samples using NanoStringDiff method

NanoString PAM50 Genes	Hs578T spiked		SkBr3 spiked		MDA-231 spiked		MCF7 spiked	
	*p*-value	*q*-value	*p*-value	*q*-value	*p*-value	*q*-value	*p*-value	*q*-value
ACTR3B	0.090	0.317	0.063	0.266	**0.001**	**0.006**	**0.010**	**0.031**
ANLN	**0.000**	**0.002**	0.018	0.167	**0**	**0**	**0**	**0.001**
BAG1	0.837	0.937	0.266	0.564	0.039	0.068	0.026	0.066
BCL2	0.647	0.887	0.821	1.000	0.125	0.173	0.221	0.334
BIRC5	0.668	0.887	0.848	1.000	0.384	0.446	0.625	0.725
BLVRA	0.529	0.854	0.835	1.000	0.080	0.122	0.040	0.092
CCNB1	0.018	0.100	0.419	0.698	**0**	**0.001**	0.023	0.061
CCNE1	0.381	0.733	0.762	1.000	0.959	1	0.905	0.923
CDC20	0.487	0.854	0.873	1.000	0.401	0.456	0.539	0.642
CDC6	0.098	0.317	0.084	0.301	**0**	**0**	1	1
CDCA1	0.024	0.119	0.323	0.621	**0**	**0.001**	**0.004**	**0.014**
CDH3	0.058	0.259	0.069	0.266	**0**	**0.002**	0.219	0.334
CENPF	0.215	0.527	0.108	0.361	0.029	0.062	0.689	0.733
CEP55	0.707	0.887	0.616	0.933	0.091	0.135	0.150	0.259
CXXC5	0.728	0.887	0.764	1.000	0.130	0.175	0.261	0.363
EGFR	0.703	0.887	0.036	0.222	0.109	0.156	0.117	0.213
ERBB2	0.599	0.887	**0.001**	**0.031**	0.031	0.064	**0**	**0.001**
ESR1	0.258	0.587	0.201	0.528	**0.001**	**0.003**	**0**	**0**
EXO1	**0.005**	**0.034**	0.242	0.549	**0.003**	**0.009**	**0.009**	**0.029**
FGFR4	0.807	0.937	0.897	1.000	0.081	0.122	0.311	0.421
FOXA1	**0.001**	**0.010**	1	1	0.033	0.064	**0**	**0.001**
FOXC1	0.713	0.887	0.056	0.266	0.252	0.317	0.676	0.733
GPR160	0.529	0.854	0.985	1.000	0.743	0.808	0.487	0.608
GRB7	0.574	0.887	0.008	0.130	1	1	0.039	0.092
KIF2C	0.309	0.648	0.271	0.564	**0**	**0**	**0**	**0**
KNTC2	0.711	0.887	0.220	0.528	**0.012**	**0.029**	0.254	0.363
KRT14	**0**	**0**	0.696	0.995	**0.015**	**0.035**	0.022	0.061
KRT17	**0**	**0**	1	1	0.375	0.446	0.526	0.642
KRT5	0.848	0.937	0.189	0.528	0.038	0.068	0.060	0.126
MAPT	0.074	0.286	0.022	0.167	0.074	0.122	0.189	0.305
MDM2	0.222	0.527	0.040	0.222	**0**	**0.001**	**0.002**	**0.008**
MELK	0.426	0.789	0.294	0.588	**0.012**	**0.029**	0.653	0.725
MIA	0.311	0.648	0.215	0.528	0.618	0.687	0.117	0.213
MKI67	0.977	1.000	0.639	0.940	0.033	0.064	0.080	0.160
MLPH	**0.007**	**0.043**	**0**	**0**	**0**	**0**	**0**	**0**
MMP11	0.862	0.937	0.195	0.528	0.253	0.317	0.160	0.266
MYBL2	**0.002**	**0.016**	1	1	**0.002**	**0.007**	**0**	**0.001**
MYC	0.513	0.854	0.819	1.000	0.299	0.365	0.451	0.579
ORC6L	0.671	0.887	0.599	0.933	**0.001**	**0.003**	**0.002**	**0.007**
PGR	0.151	0.444	0.222	0.528	0.039	0.068	0.332	0.436
PHGDH	0.101	0.317	0.019	0.167	**0.003**	**0.009**	**0.011**	**0.033**
PTTG1	0.187	0.492	0.069	0.266	0.077	0.122	0.230	0.339
RRM2	0.757	0.901	0.961	1.000	0.979	1	0.873	0.910
SFRP1	0.377	0.733	0.417	0.698	**0.006**	**0.016**	0.119	0.213
SLC39A6	0.933	0.992	0.391	0.698	0.159	0.209	**0**	**0**
TMEM45B	1	1	0.023	0.167	1	1	0.652	0.725
TYMS	**0.003**	**0.025**	0.547	0.883	**0**	**0**	**0**	**0.001**
UBE2C	0.179	0.492	0.746	1.000	**0.002**	**0.007**	**0.002**	**0.008**
UBE2T	0.062	0.259	1	1	**0**	**0**	**0.001**	**0.003**

Significant *p*-values and *q*-values <0.05. Values in bold correspond to statistically significantly expressed genes among spiked samples by NanoStringDiff analysis. Gray boxes highlight the genes found differentially expressed by both nCounter and NanoStringDiff analyses.

## DISCUSSION

Circulating tumor cells have enormous potential as a liquid biopsy of the real-time status of tumor biology without *a priori* knowledge of what tumor markers may be present. This very fact allows CTCs to serve as a potential target discovery tool but leaves the approach vulnerable to misinterpretation due to confounding by background peripheral blood leukocytes. The objective of our study was to evaluate the feasibility and accuracy of using the NanoString PAM50 Research-Use-Only code set for gene expression profiling of breast cancer CTCs isolated using the ANGLE Parsortix system. While we have optimized a workflow for transcriptomic profiling of rare CTCs using the Parsortix system, in this report, our data indicate that the NanoString PAM50 panel has limited utility in differentiating CTC mimics from leukocyte background.

In our hands, capture rates for spiked cell line CTC mimics ranged from 61-75% with the Parsortix system, which is similar to other investigators’ reports [8, 14]. Our group previously reported that CTC recovery rates using EpCAM based selection with FACS sorting were highly dependent on the intrinsic subtype of the cell line tested, with claudin-low cells showing the lowest recovery rates [17, 18]. Based on those findings and the lack of a priori knowledge of specific surface markers when processing breast cancer liquid biopsies, we sought to use an epitope independent CTC enrichment platform to overcome the limitation in isolating claudin low and/or EpCAM negative cells. The Parsortix system is advantageous over FACS in that it does not rely on affinity-based selection and permits harvesting isolated CTCs within 90 minutes of blood draw via a bench top assay, which is advantageous both logistically and in terms of RNA quality of harvested cells. The Parsortix isolates could then be used to obtain single cells using other technologies, however, this would add hours to the process and would no doubt degrade the RNA. The Parsortix harvests are not ultrapure populations of CTCs, therefore, it is important to consider the possibility of overlap in gene expression between cancer cells and leukocytes for genes of interest empirically known to be relevant to breast cancer. Therefore, when working with enriched but not ultra-pure CTC samples, amplified gene expression of background leukocytes may influence read counts. Hence, different or additional normalization strategies are required to eliminate background leukocyte signal for accurate gene expression profiling of CTCs. Background subtraction or normalization to PB controls is not possible in the context of NanoString PAM50 MTE since it produces inconsistent false positive results.

The NanoString PAM50 code set has been shown by others to be useful for classifying breast cancers into intrinsic subtypes and in generating risk of recurrence scores (ROR) [23, 25]. The NanoString single cell protocol calls for a multiple target enrichment step that is probe specific and involves limited amplification of cDNA (fewer than 16 cycles) to attempt to avoid amplification bias and allow for multiplexed hybridization based profiling of even single cells. The target cDNA molecules bind to barcode and capture probes resulting in detectable fluorescent spots [26]. The number and type of each fluorophore barcode is counted [27]. NanoString assays without MTE were previously reported to have an assay sensitivity of less than one copy of mRNA per cell, which is superior to microarrays and on par with QPCR in sensitivity [28]. One of the main advantages of NanoString in general is that it does not typically require conversion of RNA to cDNA or MTE, however, those steps are essential when performing rare cell profiling such as for characterizing CTCs. Although PCR based amplification prior to barcode capture is necessary to obtain robust read counts, our findings indicate that it may introduce false positive results and may introduce errors that lead to generating sequence changes not present in the original sample (primer dimer, DNA polymerase error, etc.) [29]. Therefore we emphasize that additional calibration/normalization steps and correction factors for pre-amplification bias are essential for single cell gene expression analysis of nCounter NanoString data. Moreover, as demonstrated in this paper, the inconsistent non-linear target amplification during MTE steps produced false positive results hindering the use of unspiked control gene expression as background (to eliminate leukocyte gene signal) that could be subtracted from spiked samples when evaluating for a unique CTC gene expression signature based on the spiked samples. This has relevance to potential future use in clinical trials to ensure that patients are not incorrectly classified by gene expression given the observed biases.

In this report, we utilized two types of negative controls – bulk, unsorted peripheral blood from healthy donors, and a second tube of blood from each donor that was processed on the Parsortix to control for the effect of size based microfluidics selection on leukocyte background gene expression (unspiked samples). CTC spike in mimics were also compared to a positive control of known cell lines. Had we not included the unspiked control samples in our study, we would have erroneously concluded that our principle component analysis of NanoString PAM50 data provided conclusive evidence that CTCs could be separated from peripheral blood in terms of gene expression profiling with the PAM50 (Figure 3). Spiked and unspiked Parsortix processed specimens clustered more closely together than with either peripheral blood or cell line controls. Multiple target enrichment clearly influenced transcript count results by consistently yielding false positive results (Figures 4 and 5). We did not observe these same issues with false positives in our ongoing RNA-seq studies of breast cancer samples (data not shown), which argues against the alternate hypothesis that it is the Parsortix system's microfluidics filtration that introduces bias to gene expression. Moreover, lack of correlation of the gene expression between the positive controls (cell line and ‘gold standard’ controls) cautions against performing targeted PCR amplification prior to gene expression analysis unless a method is validated to not introduce high false positive calls.

This study emphasizes the importance of either selecting genes that are entirely not expressed in peripheral blood or of performing a careful background subtraction or normalization procedure that considers the peripheral blood gene expression signature that is unique to each patient given that considerable heterogeneity is present both in CTCs and in the peripheral blood specimens from patient to patient. In actual cancer patients, no positive control exists to reliably distinguish signal from noise.

We concluded that the NanoString recommended targeted pre-amplification of the PAM50 Research-Use-Only code set introduced false positive results due to pre-amplification bias, and the amplification of non-cancer genes from normal leukocytes confounded gene expression profiling of CTCs. Pre-amplification bias is a concern for other similar assays that may be used as discovery tools or target validation of transcripts of interest in gene expression profiling of CTCs. We recommend the use of an unspiked negative control when evaluating CTC technologies regarding gene expression profiling.

The ANGLE Parsortix has broad potential applications in CTC research given that it is capable of rapidly capturing CTCs without a requirement for affinity based marker selection. Our workflow for whole transcriptome RNA Seq profiling with the Parsortix is the basis for ongoing clinical trials at our institution. However, the current study suggests that target validation with the NanoString PAM50 is not a viable option for CTC research.

## MATERIALS AND METHODS

### Blood specimens and cell lines

By standard venipuncture procedure, two blood samples of 7.5ml each from 19 different cancer-free female donors were collected in EDTA tubes (Becton Dickinson, Franklin Lakes, NJ). Donors were selected based on the inclusion criteria established in the approved IRB protocol. Adhering to institutional HIPPA regulations, female donors between 25 and 50 years of age signed informed consent, and a research numerical identifier was assigned to each participant to keep demographic information blinded from the researchers.

One tube of blood was used as a CTC mimic by adding or spiking in the basal-like/triple-negative breast cancer cell lines Hs578T and MDA-MB-231, or the HER2 amplified SkBr3, or the luminal A cell line MCF7. Equal numbers of blood samples (n=6) were spiked with each cell line. Some donors consented for repeated blood draws; therefore some of them were spiked with more than one cell line. These CTC mimic specimens were termed spiked samples. The spiking in process was performed within 20 minutes after blood collection. The other blood specimens were used as non-CTC or negative controls, termed unspiked controls (Figure 7).

All cell lines were grown at 37°C with 5% CO_2_. Hs578T (ATTC HTB-126) and MDA-MB-231 (ATTC HTB-26) cells were grown in complete DMEM-Dulbecco's Modified Eagle's Medium supplemented with 10% of FBS (fetal bovine serum). SkBr3 (ATCC HTB-30) was grown in McCoy's 5A Modified Medium with 10% FBS, and MCF7 (ATCC HTB-22) cells were cultured in RPMI 1640 Medium with 10% FBS. Cells were typsinized, manually counted, and a total of 20 cells were spiked into each tube of blood.

All cell lines were tested for Mycoplasma contamination using the MycoAlert *Mycoplasma* detection kit (Lonza, Walkersville, MD), and subjected to short tandem repeat genotyping for cell line authentication at the University of Arizona Genetics Core.

### CTC enrichment

Both spiked samples and unspiked controls were subjected to CTC enrichment using the ANGLE Parsortix System (ANGLE plc, Guildford, UK). Parsortix cell-harvests were recovered in 200μl of 2% bovine serum albumin BSA (Sigma-Aldrich, St. Louis. MO) in PBS. Recovered cells were centrifuged at 400xg for 5 minutes at ambient temperature, and the cell pellet was lysed in 5μl of the Prelude Direct Lysis Module (NuGEN, San Carlos, CA). Cell lysates were stored at −80°C.

### Enumeration of harvested CTCs by flow cytometry

To 7.5 ml of peripheral blood we spiked 20 SkBr3 cells and performed CTC enrichment using the Parsortix system as described above. CTC harvested material was incubated with 1μl of FITC mouse anti-human EpCAM IgG (BD Biosciences, San Jose, CA) and 1μl of PE-Cy7 mouse anti-human CD45 IgG (BD Biosciences, San Jose, CA) on ice for 30 minutes. Unbound antibodies were removed by washing twice with 2ml of wash buffer (2% BSA in PBS). Cells were resuspended in 200μl of wash buffer and analyzed using a FACS Aria II Cytometer (BD Biosciences, San Jose, CA). Cells were sorted and collected in 5μl of cell lysis buffer, and stored at −80°C.

### NanoString PAM50 nCounter single cell gene expression assay

NanoString nCounter gene expression assay has been previously described [19, 23]. Briefly, NanoString PAM50 nCounter assay is a hybridization technique that quantitatively measures the number of mRNA transcripts using a pair of target-specific multiplexed CodeSet probes (capture and reporter probes) (NanoString Technologies, Seattle, WA). The capture probe consists of a 5’ to 3’, 30-50-base target specific sequence following two 15-base sequences common to all capture probes, and a biotin affinity tag. The reporter probe consists of a 3’ to 5’, 30-50-base target specific sequence that hybridizes near to the capture probe-complementary-site, followed by 4 tandem repeats of 15 bases common to all reporter probes, and a color-coded barcode DNA/RNA hybrid molecule labeled with a fluorescent dye. After hybridizing the capture and reporter probes to the target mRNA, those mRNA/reporter/capture probe complexes were captured and immobilized via a streptavidin-biotin linkage to the surface of an nCounter cartridge. Cartridges were transferred to the nCounter Digital Analyzer (NanoString Technologies, Seattle, WA) for imaging and data collection. The expression level of a gene was measured by counting the number of times a specific fluorescent barcode molecule was detected. Counts were tabulated, exported and analyzed using internal hybridization negative and positive controls.

NanoString PAM50 targeted single cell gene expression assays were performed on CTC enriched cell lysates from both spiked samples and unspiked controls. NanoString standard protocol for single cell gene expression analysis consisted of a two-step process: cDNA conversion and multiplexed target enrichment (MTE). Initially, input RNA material (5μl of cell lysate) was converted to cDNA by reverse transcription using 1.5μl of the SuperScript VILO Master Mix (Invitrogen, Carlsbad, CA). The reaction was incubated at 25°C for 10 minutes, followed by 42°C for 60 minutes and 85°C for 5 minutes in a T-100 Thermal Cycler (BioRad, Hercules, CA). Obtained cDNA was PCR amplified using 7.5μl of TaqMan PreAmp Master Mix (Applied Biosystems, Foster City, CA) and 1μl of a pool of PAM 50 MTE primers (Table 2). The amplification process followed a denaturation step of 94°C for 10 minutes, 14 MTE cycles of 94°C for 15 seconds and 60°C for 4 minutes. MTE primer pool allowed specific linear amplification of the 56 genes included in the NanoString PAM50 CodeSet.

### Positive and negative controls for NanoString PAM50 gene expression

As NanoString PAM50 single cell gene expression positive controls, RNA from all cell lines was included. Cell line RNA extraction process included cell lysis using TRIzol (Invitrogen, Carlsbad, CA), RNA precipitation with isopropanol (Sigma-Aldrich, St. Louis, MO), and RNA wash with 70% ethanol (Sigma-Aldrich, St. Louis, MO). Isolated RNA was eluted in RNAase-free water, and both quantity and purity were access by spectrometry using NanoDrop 2000 (Thermo Scientific, Carlsbad, CA). A total of 0.1ng of cell line RNA was used for the NanoString PAM50 steps of cDNA synthesis and MTE applying similar conditions used on the spiked samples and the unspiked controls. Additionally, as ‘gold standard’ gene expression positive control we used 100ng of RNA from the cell lines. Neither cDNA conversion nor MTE steps were performed on the ‘gold standard’ control.

Peripheral blood (PB) RNA extracted from each unspiked blood specimen was included as negative gene expression control. Immediately after blood draw, 200μl of peripheral blood was stabilized by adding 1ml of RNA*later* solution (Ambion, Carlsbad, CA), and stored at −80°C. PB RNA was isolated using the RiboPure-Blood Kit (Ambion, Carlsbad, CA), and 100ng of it was used as input material for the NanoString PAM50 assay. These specimens were termed bulk peripheral blood controls (bulk PB controls) (Figure 7).

Due to the high sensitivity and high specificity of the NanoString PAM50 platform in FFPE, assays on both samples and controls were performed a single time, without technical replicates, per manufacturer's recommendations even for rare samples.

### Data normalization and differential gene expression analysis

nCounter data was analyzed using NanoString nSolver Analysis Software v2.5. Following NanoString's recommended protocol, raw count data normalization included: (i) quality control measurements for imaging quality and sample binding saturation using hybridization positive controls, (ii) elimination of experimental variability using reference genes, and (iii) per sample background correction by setting a gene expression threshold or cut-off value of 2 standard deviations over the mean of the internal hybridization negative controls. Log_2_-transformed data was used for ANOVA testing to compare the mean of the gene expression between all unspiked controls and all peripheral blood RNA samples.

Differential gene expression analysis between spiked samples and unspiked controls was initially performed by manual comparison of the background corrected Log_2_ gene expression values per gene. Results were subsequently validated using the NanoStringDiff statistical method for gene expression analysis described by Wang, et al [24]. ANOVA testing was done using GraphPad Prism 7 (La Jolla, CA).

## Abbreviations

ANOVA: Analysis of variance
CD45: Protein tyrosine phosphatase, receptor type, C
cDNA: Complementary DNA
CTCs: Circulating Tumor Cells
DNA: Deoxyribonucleic acid
EDTA: Ethylenediaminetetraacetic acid
EMT: Epithelial to Mesenchymal Transition
EpCAM: Epithelial Cell Adhesion Molecule
FACS: Fluorescence Activated Cell Sorting
GFP: Green Fluorescent Protein
HER2: Human Epidermal Growth Factor Receptor 2
mRNA: Messenger RNA
MTE: Multiple Target Enrichment
PAM50: Prediction Analysis of Microarray 50
PB: Peripheral blood
PCR: Polymerase Chain Reaction
qPCR: quantitative Polymerase Chain Reaction
RNA: Ribonucleic acid
RNA: Seq RNA sequencing
WBC: White blood cells

## References

[1] Alix-Panabieres C, Pantel K (2016). Clinical applications of circulating tumor cells and circulating tumor DNA as liquid biopsy. Cancer Discov.

[2] Alix-Panabieres C, Pantel K (2014). Challenges in circulating tumour cell research. Nat Rev Cancer.

[3] Krebs MG, Hou JM, Ward TH, Blackhall FH, Dive C (2010). Ciculating tumour cells: their utility in cancer management and predicting outcomes. Ther Adv Med Oncol.

[4] Castle J, Shaker H, Morris K, Tugwood JD, Kirwan CC (2014). The significance of circulating tumour cells in breast cancer: a review. Breast.

[5] Forte VA, Barrak DK, Elhodaky M, Tung L, Snow A, Lang JE (2016). The potential for liquid biopsies in the precision medical treatment of breast cancer. Cancer Biol Med.

[6] Pantel K, Alix-Panabieres C (2016). Functional studies on viable circulating tumor cells. Clin Chem.

[7] Sieuwerts AM, Mostert B, Bolt-de Vries J, Peeters D, de Jongh FE, Stouthard JM, Dirix LY, van Dam PA, Van Galen A, de Weerd V, Kraan J, van der Spoel P, Ramirez-Moreno R (2011). mRNA and microRNA expression profiles in circulating tumor cells and primary tumors of metastatic breast cancer patients. Clin Cancer Res.

[8] Chudziak J, Burt DJ, Mohan S, Rothwell DG, Mesquita B, Antonello J, Dalby S, Ayub M, Priest L, Carter L, Krebs MG, Blackhall F, Dive C (2016). Clinical evaluation of a novel microfluidic device for epitope-independent enrichment of circulating tumour cells in patients with small cell lung cancer. Analyst.

[9] Lustberg M, Jatana KR, Zborowski M, Chalmers JJ (2012). Emerging technologies for CTC detection based on depletion of normal cells. Recent Results Cancer Res.

[10] Alix-Panabieres C, Pantel K (2013). Circulating tumor cells: liquid biopsy of cancer. Clin Chem.

[11] Alix-Panabieres C, Pantel K (2014). Technologies for detection of circulating tumor cells: facts and vision. Lab Chip.

[12] Joosse SA, Gorges TM, Pantel K (2015). Biology, detection, and clinical implications of circulating tumor cells. EMBO Mol Med.

[13] Gorges TM, Kuske A, Rock K, Mauermann O, Muller V, Peine S, Verpoort K, Novosadova V, Kubista M, Riethdorf S, Pantel K (2016). Accession of tumor heterogeneity by multiplex transcriptome profiling of single circulating tumor cells. Clin Chem.

[14] Hvichia GE, Parveen Z, Wagner C, Janning M, Quidde J, Stein A, Muller V, Loges S, Neves RP, Stoecklein NH, Wikman H, Riethdorf S, Pantel K (2016). A novel microfluidic platform for size and deformability based separation and the subsequent molecular characterization of viable circulating tumor cells. Int J Cancer.

[15] Xu L, Mao X, Imrali A, Syed F, Mutsvangwa K, Berney D, Cathcart P, Hines J, Shamash J, Lu YJ (2015). Optimization and evaluation of a novel size based circulating tumor cell isolation system. PLoS One.

[16] Ring A, Forte V, Barrak D, Porras T, Punj V, Carrasco S, Yu M, Tripathy D, Lang JE (2016). Abstract 1549: Molecular profiling of circulating tumor cells as a surrogate for distant metastasis in stage IV breast cancer. Cancer Res.

[17] Lang JE, Scott JH, Wolf DM, Novak P, Punj V, Magbanua MJ, Zhu W, Mineyev N, Haqq CM, Crothers JR, Esserman LJ, Tripathy D, van ’t Veer L (2015). Expression profiling of circulating tumor cells in metastatic breast cancer. Breast Cancer Res Treat.

[18] Ring A, Mineyev N, Zhu W, Park E, Lomas C, Punj P, Yu M, Barrak D, Forte V, Porras T, Tripathy D, Lang JE (2015). EpCAM based capture detects and recovers circulating tumor cells from all subtypes of BrCa except claudin-low. Oncotarget.

[19] Geiss GK, Bumgarner RE, Birditt B, Dahl T, Dowidar N, Dunaway DL, Fell HP, Ferree S, George RD, Grogan T, James JJ, Maysuria M, Mitton JD (2008). Direct multiplexed measurement of gene expression with color-coded probe pairs. Nat Biotechnol.

[20] Gnant M, Filipits M, Greil R, Stoeger H, Rudas M, Bago-Horvath Z, Mlineritsch B, Kwasny W, Knauer M, Singer C, Jakesz R, Dubsky P, Fitzal F (2014). Predicting distant recurrence in receptor-positive breast cancer patients with limited clinicopathological risk: using the PAM50 Risk of Recurrence score in 1478 postmenopausal patients of the ABCSG-8 trial treated with adjuvant endocrine therapy alone. Ann Oncol.

[21] Sestak I, Cuzick J, Dowsett M, Lopez-Knowles E, Filipits M, Dubsky P, Cowens JW, Ferree S, Schaper C, Fesl C, Gnant M (2015). Prediction of late distant recurrence after 5 years of endocrine treatment: a combined analysis of patients from the Austrian breast and colorectal cancer study group 8 and arimidex, tamoxifen alone or in combination randomized trials using the PAM50 risk of recurrence score. J Clin Oncol.

[22] Filipits M, Nielsen TO, Rudas M, Greil R, Stoger H, Jakesz R, Bago-Horvath Z, Dietze O, Regitnig P, Gruber-Rossipal C, Muller-Holzner E, Singer CF, Mlineritsch B (2014). The PAM50 risk-of-recurrence score predicts risk for late distant recurrence after endocrine therapy in postmenopausal women with endocrine-responsive early breast cancer. Clin Cancer Res.

[23] Parker JS, Mullins M, Cheang MC, Leung S, Voduc D, Vickery T, Davies S, Fauron C, He X, Hu Z, Quackenbush JF, Stijleman IJ, Palazzo J (2009). Supervised risk predictor of breast cancer based on intrinsic subtypes. J Clin Oncol.

[24] Wang H, Horbinski C, Wu H, Liu Y, Sheng S, Liu J, Weiss H, Stromberg AJ, Wang C (2016). NanoStringDiff: a novel statistical method for differential expression analysis based on NanoString nCounter data. Nucleic Acids Res.

[25] Prat A, Fan C, Fernandez A, Hoadley KA, Martinello R, Vidal M, Viladot M, Pineda E, Arance A, Munoz M, Pare L, Cheang MC, Adamo B (2015). Response and survival of breast cancer intrinsic subtypes following multi-agent neoadjuvant chemotherapy. BMC Med.

[26] Khodakov D, Wang C, Zhang DY (2016). Diagnostics based on nucleic acid sequence variant profiling: PCR, hybridization, and NGS approaches. Adv Drug Deliv Rev.

[27] Goodwin S, McPherson JD, McCombie WR (2016). Coming of age: ten years of next-generation sequencing technologies. Nat Rev Genet.

[28] Kulkarni MM (2011). Digital multiplexed gene expression analysis using the NanoString nCounter system. Curr Protoc Mol Biol.

[29] Peng Q, Vijaya Satya R, Lewis M, Randad P, Wang Y (2015). Reducing amplification artifacts in high multiplex amplicon sequencing by using molecular barcodes. BMC Genomics.

